# High-throughput screening reveals novel mutations in spinal muscular atrophy patients

**DOI:** 10.1186/s13052-020-00925-1

**Published:** 2020-11-04

**Authors:** Ruiping Zhang, Chunyu Gu, Linjie Pu, Yingtao Meng, Jianbo Shu, Chunquan Cai

**Affiliations:** 1 Department of Pediatric, Tianjin Children’s Hospital, Tianjin, 300134 China; 2 Graduate School, Tianjin Medical University, Tianjin, 300070 China; 3 Tianjin Pediatric Research Institute, Tianjin Children’s Hospital, 238 Longyan Road, Beichen District, Tianjin, 300134 China; 4 Tianjin Key Laboratory of Birth Defects for Prevention and Treatment, Tianjin, 300134 China; 5 Department of Neurosurgery, Tianjin Children’s Hospital, 238 Longyan Road, Beichen District, Tianjin, 300134 China

**Keywords:** Spinal muscular atrophy (SMA), High-throughput sequencing, SMN1, Onset

## Abstract

**Background:**

Spinal muscular atrophy (SMA) is an autosomal recessive hereditary disease associated with severe muscle atrophy and weakness in the limbs and trunk. The discovery of mutated genes is helpful in diagnosis and treatment for SMA.

**Methods:**

Eighty-three whole blood samples were collected from 28 core families of clinically suspected SMA, and multiplex ligation probe amplification (MLPA) was performed. Afterwards, the complete gene sequence of *SMN1* gene was detected. Furthermore, 20 SMA patients were selected from the 28 probands, and 5 non SMA children as controls. The Life Technologies SOLiD™ technology with mate-pair chemistry was utilized to conduct the whole exome high-throughput sequencing.

**Results:**

Twenty-two probands were SMA patients, 3 probands carriers, and 3 probands normal individuals. Moreover, 2 parents from 2 SMA families were with 3 *SMN1* exon7 copies. Six *SMN1* single nucleotide variants (SNVs) were identified in the 83 samples, and c.[84C > T], c.[271C > T], c.[−39A > G] and g.[70240639G > C] were novel. Compared with control group, 9102 mutation were selected out in SMA patients. *SPTA1* mutation c.[−41_-40insCTCT], *FUT5* SNV c.[1001A > G], and *MCCC2* SNV c.[−117A > G] were the 3 most frequent mutations in SMA group (95, 85 and 75%, respectively).

**Conclusions:**

We identified some mutations in both *SMN1* and other genes, and c.[271C > T], c.[−41_-40insCTCT], c.[1001A > G] and c.[−117A > G] might be associated with the onset of SMA.

## Background

Spinal muscular atrophy (SMA) is an autosomal recessive hereditary disease characterized by degeneration of spinal cord motor neurons, atrophy of skeletal muscles, and generalized weakness [1]. It affects 1 in 10,000 live births, and often leads to early death [2]. SMA manifests over a wide range of severity, affecting infants through adults. According to the onset time and severity of the disease, SMA is divided into 4 types (SMA1, SMA2, SMA3 and SMA4), and SMA 1, with onset before age 6 months; SMA 2, with onset between age 6 and 18 months; SMA 3, with onset in childhood after age 12 months; and SMA 4, with adult onset [3]. Nusinersen (trade name: Spinraza) is the only approved drug to treat spinal muscular atrophy, which is administered directly to the central nervous system using an intrathecal injection [4], but it’s mandatory to have a specific diagnosis in quick time. The genetic profile is essential for the quick and accurate diagnosis of SMA.

SMA is caused by homozygous disruption of the survival motor neuron 1 (*SMN1*) gene by deletion, conversion, or mutation [1]. Since SMA is one of the most common lethal genetic disorders, with a carrier frequency of 1 in 40 to 1 in 60, direct carrier dosage testing has been beneficial to many families [5]. About 96% SMA are caused by a homozygous deletion of *SMN1* exon7, and the remaining 4% of cases are caused either by compound heterozygosity with a point mutation in one allele and a deletion in the other or by compound heterozygous point mutations in *SMN1* [6]. *SMN2* is a homologous gene of *SMN1* and functions as a SMA modifier. In general, the copy number of *SMN2* is substantial variation in SMA patients, and a high *SMN2* copy number tends to a milder type [7]. Furthermore, more and more new genes or novel mutations have been reported to be related to the morbidity, severity, treatment and prognosis of SMA with the development of gene sequencing technology. A study revealed seven different mutations of *SMN1*, and among them, c.824G > C, and c.825-2A > T were described for the first time [8]. Another study found the *NAIP* copy number was inversely correlated with the clinical severity of SMA [9]. *GTF2H2* and *H4F5* have been proved to be associated with the onset and type of SMA [10]. It is important to understand the genetic characteristics of SMA pathology in order to better understand and diagnose it, so as to implement the nursing care of more and more affected patients.

The severity of SMA varies from infancy to adulthood, which is reflected in the clinical classification system of type 1–4. In addition, a recent study reported that the copy number of *SMN* genes may determine the pathological type of SMA patients. Cao et al. [11] found that in different copy numbers of *SMN2*, the distribution of type I and type 2/3 was significantly different (*P* < 0.001): when the copy number of *SMN2* in parents was 1, 75% of SMA progenies were type 1 and 25% were type 2/3. However, when the parents carried three copies of *SMN2*, their SMA progenies were all 2/3 type. Therefore, it is very important to understand the pathological genetic characteristics of patients, which directly affects the diagnosis and treatment of patients.

To further improve our understanding of genetic variation in patients with SMA, in this study, multiplex ligation probe amplification (MLPA) was firstly used for preliminary diagnosis in 28 core families of suspected SMA patients, and then the complete gene sequence of *SMN1* gene was detected by high-throughput sequencing to find more mutations in the 28 core families. Afterwards, 20 children diagnosed with SMA and 5 children diagnosed with non SMA were enrolled, and the whole exome screening of other related genes was performed to explore more genes and mutations involved in the onset of SMA.

## Methods

### Patients and samples

From December 2013 to May 2017, 28 probands of clinically suspected SMA, 15 males and 13 females, were accepted by our hospital (Tianjin Children’s Hospital, Tianjin, China) because of unstable walking, and their age ranging from 1 month to 12 years. The phenotypes of their parents were normal. 3–5 ml peripheral blood samples from the 83 enrolled cases (probands and their parents) were collected. All the subjects signed the informed consent forms for genetic testing routinely, and all procedures were in accordance with the ethical standards of the institutional and/or national research committee.

### MLPA

Extracted Genomic DNA with the salting out method. The nucleic acid quantitative instrument NADO DROP 2000 (Thermo Fisher Scientific Inc., Waltham, USA) was utilized to determine the quality and quantity of the extracted DNA. MLPA was performed using a SALSA MLPA Kit P021 (MRC-Holland, Amsterdam, Netherlands) according to the manufacturer’s protocol. MLPA products were run on an ABI PRISM 3130 genetic analyzer (Applied Biosystems International Inc., California, USA). The evaluation criteria were based on the kit instructions: in normal individuals, SMN1 exon7 is 2 copies; the *SMN1* gene of patients with homozygous deletion and carriers with heterozygous deletion is 0 copies and 1copy, respectively [12, 13].

### SMN1 gene screening by high-throughput sequencing

The complete genome sequence of *SMN1* gene was detected from above 83 enrolled cases via high-throughput sequencing. Firstly, high-molecular-weight genomic DNAs were extracted with the DNeasy Blood and Tissue kit (QIAGEN, Dusseldorf, Germany) and 10 μg genomic DNA was used for library generation according to the manufacturer’s recommendations. Secondly, the Illumina HiSeq-2500 platform (Illumina, California, USA) was used to conduct the sequencing reaction based on the manufacturer’s protocol. The average sequencing depth was 100×, and more than 96% regions was up to 20×. Thirdly, the raw data were compared with human genome (NCBI build 36, hg18), and marked repeated reads and filtered out low-quality data. Fourthly, the base quality of reads was calibrated again by the genome analysis toolkit (GATK) algorithm, and ultimately all the a single base of DNA (point mutation) or a loss of base pairs (deletion) were screened out with the GATK software V3.0 (Eli and Edythe L. Broad Institute, Massachusetts, USA) [14]. The QC_30_ of the raw data was more than 85% in all samples, and the allele frequency of all mutations was more than 20%.

### The whole exome screening by high-throughput sequencing

Based on previous results, 20 SMA patients with homozygous deletion of *SMN1* exon7 were selected from the 28 probands, and 5 non SMA children were as controls (2 children carried with heterozygous deletion of *SMN1* exon7 and 3 children with 2 *SMN1* exon7 copies). Whole exome high-throughput sequencing was performed by The Life Technologies SOLiD™ (version 3) technology with mate-pair chemistry. 20 μg high-molecular-weight genomic DNAs was used for library generation. The raw data were analyzed with GATK software. The average sequencing depth was 100×, and more than 96% regions was up to 20×. The QC_30_ of the raw data was more than 85% in all samples, and the allele frequency of all mutations was more than 40%.

## Results

### MLPA

MLPA results showed that 22 probands were with homozygous deletion of *SMN1* exon7 (SMA patients), 3 probands carried with heterozygous deletion of *SMN1* exon7 (carriers), and 3 probands with 2 *SMN1* copies (normal individuals). However, the mother of a SMA patient had 3 *SMN1* exon7 copies and the father was carrier, and the father of another SMA patient had 3 *SMN1* exon7 copies and the mother was carrier. Here, we recorded the above 2 SMA patients as Proband-1 and Proband-2, respectively. Furthermore, 1 carrier, a 2-year old girl, had some clinical features that correspond to SMA, such as atrophy of skeletal muscles, generalized weakness and extensive neurogenic injury by electromyography. Here, the carrier was named as Proband-3. Besides, the father of Proband-3 was without heterozygous deletion of *SMN1* exon7 and exon8, and the mother was a carrier.

### Novel mutations of SMN1 in suspected SMA family

A total of 6 single nucleotide variants (SNVs) of SMN1 were identified in the 83 samples, and they were showed in Table 1. Three SNVs located in exon, 1 in UTR5 and 2 in intron; c.[84C > T], c.[271C > T], c.[−39A > G] and g.[70240639G > C] were firstly reported here; c.[84C > T] and c.[462A > G] were synonymous mutations, and c.[271C > T] were stop-gain; c.[271C > T] caused changes in encoded amino acids. Furthermore, c.[271C > T] was found in Proband-3 and her father, and c.[462A > G] was occurred in 17 SMA patients, 2 carrier and 2 normal individuals. In addition, 1 carrier was with c.[84C > T], 1 SMA patient with c.[−39A > G], and 1 SMA patient with g.[70240639G > C].

**Table 1:** Mutations in *SMN1*

Position	MT	Ex-In	Function	REF > ALT	dbSNP	cHGVS	AAChange	Frequency
70,234,668	snv	exon2	synonymous	C > T	NA	c.[84C > T]	NA	3.61% (3/83)
70,220,892	snv	exon3	stop-gain	C > T	NA	c.[271C > T]	p.[Gln91X]	2.41% (2/83)
70,238,373	snv	exon4	synonymous	A > G	rs4915	c.[462A > G]	NA	83.13% (69/83)
70,220,892	snv	UTR5	unknown	A > G	NA	c.[−39A > G]	NA	4.82% (4/83)
70,240,639	snv	intron	unknown	G > C	NA	g.[70240639G > C]	NA	8.43% (7/83)
70,247,937	snv	intron	unknown	A > C	rs200563560	g.[70247937A > C]	NA	1.20% (1/83)

*MT* Mutation type, *Ex-In* Exon or intron, *snv* Single nucleotide variant, *NA* No report or no change, *X* Unknown amino acids, *Frequency* Frequency in all samples

### Novel mutations occurred in only SMA patients

Compared with control group, a total of 9102 mutation were selected out in SMA patients with homozygous deletion of *SMN1* exon7. They were located in the exon region, and occurred only in SMA patients not in carriers and normal individuals. Among them, 2415 genes and some indefinite genes were included, and 8619 SNVs, 267 deletions and 216 inserts were contained. Here, the indefinite genes were removed, and the 30 most frequent mutations were showed in Table 2 (Frequency ≥ 50%). It was obvious from Table 2 that only the *MCCC2* missense mutation c.[1001A > G] located on chromosome 5q13, which was the same location as *SMN1* and *SMN2*. The others were unlinked to 5q13.

**Table 2:** The 30 most frequent mutations in SMA patients

Gene	Position	MapLoc	MT	ExID	Function	cHGVS	AAChange	dbSNP	Frequency
SPTA1	158,656,347	1q23.1	ins	EX1	utr-5	c.[−41_-40insCTCT]	NA	rs111674514	95%(19/20)
FUT5	5,866,736	19p13.3	snv	EX2E	missense	c.[1001A > G]	p.[His334Arg]	rs778984	85%(17/20)
MCCC2	70,883,136	5q13.2	snv	EX1	utr-5	c.[−117A > G]	NA	rs11746722	75%(15/20)
ARL14EP	30,352,473	11p14.1	ins	EX2	utr-5	c.[−23_-22insAG]	NA	rs75725983	70%(14/20)
GNS	65,146,532	12q14.3	snv	EX2	coding-synon	c.[198G > A]	NA	rs1147096	65%(13/20)
COL3A1	189,864,080	2q32.2	snv	EX30	missense	c.[2092G > A]	p.[Ala698Thr]	rs1800255	65%(13/20)
HSPD1	198,363,504	2q33.1	snv	EX2	coding-synon	c.[69 T > C]	NA	rs1050347	65%(13/20)
SREBF1	17,714,719	17p11.2	del	EX20E	utr-3	c.[1217_1218delTT]	NA	NA	60%(12/20)
CYP2A6	41,355,849	19q13.2	snv	EX2	coding-synon	c.[217 T > C]	NA	rs2302990	60%(12/20)
SDHAP1	195,710,975	3q29	snv	IVS5	unknown	n.[750 + 8C > T]	NA	NA	60%(12/20)
LHX4	180,199,727	1q25.2	snv	EX1	coding-synon	c.[63 T > C]	NA	rs75857235	55%(11/20)
RGPD8	113,147,159	2q13	snv	EX20	coding-synon	c.[3363C > T]	NA	rs62157473	55%(11/20)
PROM1	16,020,162	4p15.32	snv	EX8	coding-synon	c.[786G > A]	NA	rs2286455	55%(11/20)
HLA-DQB2	32,726,838	6p21.32	snv	EX3	coding-synon	c.[435G > A]	NA	rs17850863	55%(11/20)
ERCC6	50,666,808	10q11.23	snv	EX21E	utr-3	c.[*53 T > C]	NA	rs4253231	50%(10/20)
NOTCH2	120,612,004	1p11.2	del	EX1	frameshift	c.[17_18delCC]	p.[Pro6Arg]	NA	50%(10/20)
GORAB	170,521,650	1q24.2	snv	EX5E	utr-3	c.[*47 T > G]	NA	rs7552922	50%(10/20)
DYNC1H1	102,493,761	14q32.31	snv	EX46	coding-synon	c.[8928A > G]	NA	rs8010870	50%(10/20)
ACSF3	89,167,138	16q24.3	snv	EX3	missense	c.[49G > C]	p.[Ala17Pro]	rs11547019	50%(10/20)
MYO5B	47,429,022	18q21.1	snv	EX21	missense	c.[2753G > A]	p.[Arg918His]	rs2298624	50%(10/20)
MYO5B	47,500,836	18q21.1	snv	EX10	coding-synon	c.[1206C > T]	NA	rs11082795	50%(10/20)
BCL10	85,742,157	1p22.3	snv	EX1	utr-5	c.[−122C > G]	NA	rs1060843	50%(10/20)
CGB5	49,547,446	19q13.33	snv	EX1	utr-5	c.[−21G > C]	NA	rs35014217	50%(10/20)
SNRNP200	96,952,833	2q11.2	snv	EX27	coding-synon	c.[3550 T > C]	NA	rs3171927	50%(10/20)
NR3C2	149,002,017	4q31.23	del	EX9E	utr-3	c.[478delA]]	NA	rs10708334	50%(10/20)
GRM1	146,755,560	6q24.3	snv	EX9E	coding-synon	c.[3213 T > G]	NA	rs1047006	50%(10/20)
RP1L1	10,467,576	8p23.1	snv	EX4E	coding-synon	c.[4032A > G]	NA	rs4840499	50%(10/20)
RP1L1	10,467,652	8p23.1	snv	EX4E	missense	c.[3956C > G]	p.[Ala1319Gly]	rs4840501	50%(10/20)
CELP	135,961,787	9q34.2	snv	EX4	ncRNA	n.[622A > G]	NA	rs10901232	50%(10/20)
CELP	135,961,796	9q34.2	snv	EX4	ncRNA	n.[631C > T]	NA	rs10901233	50%(10/20)

*MapLoc* The locations on chromosome, *MT* Mutation type, *Ex* Exon, *snv* Single nucleotide variant, *ins* Insert, *del* deletion, *NA* no report or no change, *Frequency* Frequency in the 20 SMA samples

## Discussion

Since both the clinical features and the mutation characteristics of SMA are obvious, the diagnosis of SMA is considered as relatively easy process. However, a recent review reported that the diagnostic process for SMA is not always simple, and there is usually a delay between the onset of clinical features and the diagnosis in all types of SMA [15]. At present, MLPA is the gold standard for clinical diagnosis of SMA. However, MLPA can only detect the deletion of *SMN1* according to the gene copy number, not detect point mutations of *SMN1*. It is well known that about 4% of SMA patients bear one *SMN1* copy with an intragenic mutation. Therefore, some SMA patients are inevitably misdiagnosed as carriers. In addition, the emergence of new therapies increases the need for early diagnosis of SMA, the approval of therapies and the neonatal screening programs urgently require a more detailed understanding of genetic variation [16]. In this study, Proband-3 was with one *SMN1* copy and the *SMN1* stop-gain mutation c.[271C > T], and the heterozygous deletion of *SMN1* exon 7 was from her mother, and the *SMN1* stop-gain mutation c.[271C > T] from her father. The *SMN1* stop-gain mutation c.[271C > T] was never reported before, and it led to a amino acid change. Although MLPA results showed Proband-3 to be carrier, some SMA-related clinical features were occurred on her. Here, we suspected that Proband-3 might be a SMA patient caused by the heterozygous deletion of *SMN1* exon 7 combined with the *SMN1* stop-gain mutation c.[271C > T]. Simultaneously, c.[271C > T] might be involved in the onset of SMA. In addition, [2 + 0] genotype carriers are two *SMN1* copies on one chromosome and with deletion of *SMN1* on the other chromosome [17]. In this article, we found 2 patients (Proband-1 and Proband-2) whose one parent was carriers and the other parent with 3 *SMN1* exon7 copies (Proband-1’s mother and Proband-2’s father). Based on our results we suspected that Proband-1’s mother and Proband-2’s father might be [2 + 0] genotype carriers.

*SMN1* and *SMN2* present on chromosome 5q13, and of the 5q13-linked SMA patients, 96.4% show homozygous absence of *SMN1* exons 7 and 8 or exon 7 only, whereas 3.6% present a compound heterozygosity with a subtle mutation on one chromosome and a deletion/gene conversion on the other chromosome [6]. Due to the complexity of 5q chromosome structure, the mechanism of SMA has not been fully elucidated. For example, the rare variations in *SMN2* have been described by several studies [18–20], and some scholars believe that the variations in *SMN2* locus, such as the deletion of adjacent *NAIP1* gene, will affect or even change the severity of SMA [21, 22]. With the development of bioinformatics, more and more mutations have been discovered in *SMN1*, and some of them possess significant clinical implications. Yamamoto et al. [23] revealed 4 intragenic mutations (p.Ala2Val, p.Trp92Ser, p.Thr274TyrfsX32 and p.Tyr277Cys), and location of the mutations were associated with the clinical severity of SMA. Ronchi et al. [24] described a novel *SMN1* mutation that affected the donor splice site of exon 7 and resulted in an unusually severe SMA phenotype with rapid fatal outcome in an Italian infant. In this article, we found 6 *SMN1* SNVs in 28 core families of suspected SMA patients, including 4 novel mutations c.[84C > T], c.[271C > T], c.[−39A > G] and g.[70240639G > C], which had never been previously reported. Besides, we identified more mutations combined with homozygous absence of *SMN1* exons7 (Table 2). The 3 most frequent mutations were the insertion mutation c.[−41_-40insCTCT] in *SPTA1* exon1 (rs111674514), the SNV c.[1001A > G] in *FUT5* exon2 (rs778984), and the SNV c.[−117A > G] in *MCCC2* exon1 (rs11746722). *SPTA1* encodes the human erythroid alpha-spectrin, which is an actin crosslinking and molecular scaffold protein that links the plasma membrane to the actin cytoskeleton, and functions in the determination of cell shape, arrangement of transmembrane proteins, and organization of organelles [25, 26]. Mutations in *SPTA1* can lead to a variety of hereditary red blood cell disorders, including elliptocytosis type 2, pyropoikilocytosis, and spherocytic hemolytic anemia [27, 28]. *FUT5* encodes alpha1,3-fucosyltransferase in human, the down-regulation of *FUT5* reduces the expression of sialyl-Lewis antigens and the adhesion and binding capacities of gastric cancer cells [29]. Gene transfer of alpha1,3-fucosyltransferase increased tumor growth of the PC-3 human prostate cancer cell line through enhanced adhesion to prostatic stromal cells [30]. Methylcrotonyl CoA carboxylase β (MCCβ) is encoded by *MCCC2*, and point mutations and deletion events in *MCC2* can lead to MCC deficiency [31]. MCC deficiency is a rare autosomal recessive genetic disorder whose clinical presentations range from benign to profound metabolic acidosis and death in infancy, which is has something in common with SMA in some ways. *MCCC2* locates on chromosome 5q13, which was the same as *SMN1*. Some studies indicated that *SMN1* was the causative gene, and other genes on 5q13 region acted as modifier gene (such as *SMN2*, *NAIP* and *GTF2H2*), which were associated with disease severity [32]. The mutations rs111674514 in *SPTA1*, rs778984 in *FUT5* and rs11746722 in *MCCC2* have been identified previously, but the clinical significance remains uncertain. In this article, we found they were widely prevalent in SMA patients, and almost nonexistent in non-patients. Therefore, it suggested they might be involved in the morbidity of SMA.

One limitation of this study is the failure to use healthy children as control. The reason is that the children involving in this study are younger (0 to 12 years old), many parents are reluctant to let their healthy children participate in the study because of insufficient understanding of genetic testing. Another objective reason is that the cost of sequencing is high, so parents of healthy children are unwilling to pay for it. However, in terms of the rationality of the entire study, we really should add healthy children as control to make our research more complete, and that is what we will improve in the follow-up related studies. In contrast, using non-SMA patients with similar clinical characteristics of SMA as a control could exclude some genetic mutations that may lead to similar clinical characteristics of children with SMA, so as to make the object of this study more accurate. Using this population as a control can exclude other mutations that may cause dyskinesia, and only retain mutations unique to SMA patients.

## Conclusions

We found more mutations in both *SMN1* and other genes, and some of them were associated with the onset of SMA, such as the *SMN1* stop-gain mutation c.[271C > T], the *SPTA1* insertion mutation c.[−41_-40insCTCT], the *FUT5* SNV c.[1001A > G], and the *MCCC2* SNV c.[−117A > G].

## Data Availability

The datasets used and/or analyzed during the current study are available from the corresponding author on reasonable request.

## Abbreviations

SMA: Spinal muscular atrophy
MLPA: Multiplex ligation probe amplification
SNVs: Single nucleotide variants
*SMN*: Survival of motor neuron
*NAIP*: Neuronal apoptosis inhibitory protein
GATK: Genome analysis toolkit
MCCβ: Methylcrotonyl CoA carboxylase β
